# Rebuilding lives after trauma: positive psychotherapy in the rehabilitation of PTSD patients

**DOI:** 10.1192/j.eurpsy.2026.10297

**Published:** 2026-07-31

**Authors:** A. K. Sikora

**Affiliations:** Ivano-Frankivsk National Medical University, Ivano-Frankivsk , Ukraine

## Abstract

**Abstract:**

Psychiatric rehabilitation after trauma requires more than symptom reduction; it involves restoring functional capacity, autonomy, and social participation. Refugees and forcibly displaced individuals face a high burden of PTSD and complex stressors related to loss, disrupted identity, and integration challenges, which can hinder long-term recovery. In this talk, I will present clinical reflections from psychotherapeutic work with Ukrainian and Belarusian displaced patients conducted in a private outpatient setting in Poland. I will outline how Positive Psychotherapy can be applied as an evidence-informed, culturally adaptable, strengths-based approach to rehabilitation in PTSD, focusing on resource activation, meaning-making, reconstruction of identity, and rebuilding interpersonal functioning. Particular attention will be given to the therapeutic use of narratives: how patients’ personal stories, as well as carefully framed stories of other refugees, can enhance engagement, reduce shame and helplessness, and support post-traumatic growth—while maintaining boundaries and avoiding re-traumatisation. Practical tools will be shared that can be integrated into multidisciplinary rehabilitation pathways, complementing psychoeducation, peer support, and recovery-oriented care.

**Disclosure of Interest:**

None Declared.

